# Sodium fluoride disrupts DNA methylation of *H19* and *Peg3* imprinted genes during the early development of mouse embryo

**DOI:** 10.1007/s00204-013-1122-5

**Published:** 2013-09-13

**Authors:** Jia-Qiao Zhu, Yang-Jun Si, Lai-Yang Cheng, Bao-Zeng Xu, Qi-Wen Wang, Xiao Zhang, Heng Wang, Zong-Ping Liu

**Affiliations:** 1 College of Veterinary Medicine, Yangzhou University, Wenhui Rd, Yangzhou, 225009 Jiangsu People’s Republic of China; 2 Departments of Surgery and Biology, McGill University, Montreal, QC Canada

**Keywords:** Sodium fluoride, DNA methylation, *H19*, *Peg3*, Mouse embryos

## Abstract

Sodium fluoride (NaF) is associated with embryonic and fetal development abnormalities, but the mechanism by which this occurs is unclear. DNA methylation, an important epigenetic reprogramming mechanism, is essential for normal embryonic development. Thus, we investigated the effect of NaF on DNA methylation in early mouse embryos, as well as mouse sperm and liver using bisulfite sequencing and ELISA. Data indicate that *H19*, a paternally imprinted gene, compared to control embryos, was less methylated in 8-cell embryos from pregnant mice treated with NaF (100 mg/l) in drinking water for 48 h. *Peg3*, a maternally imprinted gene, and the *Line1* repeated sequence were similarly methylated in NaF-treated and control embryos. Oral ingestion of NaF for 35 days did not significantly change *Line1* and genomic global DNA methylation in the liver. *H19*, *Rasgrf1*, *Line1,* and genomic global DNA methylation were also similar in NaF-treated and control sperm. Female mice mated with NaF-treated male mice (35 days) had less methylated *H19*, but *Peg3* was significantly more methylated. *Line1* was similarly methylated in treated 8-cell embryos, compared to control embryos. NaF treatment of male mice before copulation significantly increased the expression of *H19* in blastocysts, whereas *H19* expression was not detected in 8-cell embryos. Data suggest that NaF may interact directly with the embryo to disrupt the maintenance of normal gene imprinting during pregnancy. Long-term NaF exposure of males may not directly affect DNA methylation of the sperm and liver, but the sperm may signal to early embryos with abnormal gene imprinting.

## Introduction

Fluorine is one of the most abundant elements on the earth, and high fluoride concentrations can be found in soil and water; dental products; food and beverage products such as tea, soy products, and grape juice; and in fluorinated prescription drugs (Sun et al. 2010). Increased fluoride intake is reported to be associated with an increased prevalence of dental fluorosis. Likewise, excessive fluoride exposure can precipitate various pathological changes in reproductive tissues and in the liver (Gupta et al. 2007). Fluoride not only can cross the blood-testis barrier to inhibit testicular spermatogenesis and decrease sperm capacitation (Dvorakova-Hortova et al. 2008), but also it can readily cross the placental barrier to directly damage developing mammalian fetuses (Goh and Neff 2003). However, the mechanism of fluorine reproductive toxicity is not well understood.

DNA methylation is an important mammalian epigenetic mechanism thought to be involved in early embryonic development. Two major waves of genome-wide demethylation and remethylation occur during development: one occurs during germ cell development and the other occurs after fertilization. However, imprinted genes escape reprogramming after fertilization and retain methylation differences during development. Reprogramming is essential for normal embryonic development and this is inextricably linked with cell pluripotency (Seisenberger et al. 2012). Methylation of the *Line1* repeated sequence (10,000–100,000 copies) presumably reflects the methylation of many different genome regions. *Line1* is highly methylated overall in mature oocytes and sperm, but it is demethylated in the zygote-to-blastocyst stage (Lane et al. 2003; Okada et al. 2010).

*H19*, a paternally imprinted gene, is expressed on the maternal allele and repressed on the paternal allele. The *H19* imprint occurs during spermatogenesis, and the differentially methylated region (DMR) of *H19* is methylated in mature sperm but not in oocytes (Lucifero et al. 2002). Differential methylation of *H19* is maintained during reprogramming after fertilization and is a target for environmental insults (Joss-Moore and Lane 2012; Park et al. 2004).

The DMR of *Peg3*, a maternally imprinted gene, is unmethylated in sperm but completely methylated in mature oocytes (Lucifero et al. 2002). In a previous study, we reported that the *Peg3* methylation imprint can be erased and re-established during pre-implantation development (Liu et al. 2008). The unique methylation dynamics of *Peg3* indicate that the process of methylation imprint establishment during pre-implantation development is more complex than previously thought (Imamura et al. 2005). *Peg3* methylation errors can cause aberrant development. Recent research suggests that the *Peg3* methylation status may be a useful molecular marker for cervical intra-epithelial neoplasia (Nye et al. 2013). Thus, *Peg3* methylation also may be easily influenced by environmental factors.

This leads us to speculate that NaF may change the normal DNA methylation pattern of early embryos. Therefore, we investigated whether NaF interferes with DNA methylation, and we report that possible links exist between the DNA methylation and NaF reproductive toxicity.

## Materials and methods

### Materials and chemicals

Chemicals and media were purchased from Sigma-Aldrich (St. Louis, MO) unless specifically mentioned. NaF was purchased from Suyi Chemical Company (Shanghai, China). Pregnant mare serum gonadotropin (PMSG) and human chorionic gonadotropin (hCG) were obtained from Ningbo Second Hormone Factory (Ningbo, China). A DNA easy kit and RNeasy Micro Kit were procured from Qiagen (Germany). A MethyFlash Methylated DNA Quantification Kit was purchased from Epigentek (USA). Sodium metabisulfite was purchased from Merck (Germany). ExTaq hotstart polymerase, PrimeScript reverse transcriptase, and T vector were procured from TaKaRa Biotechnology Company (Dalian, China). Wizard SV Gel and the PCR Clean-Up System were purchased from Promega Biotech Company (Beijing, China). Restriction enzymes (TaqI) were purchased from New England Biolabs (USA).

### Ethics statement

This study was carried out in strict accordance with the recommendations of the Guide for the Care and Use of Laboratory Animals of the National Research Council. All procedures described in the present study were reviewed and approved by the Animal Care and Use Committee of Yangzhou University (approval ID: SYXK (Su) 2007-0005). Healthy ICR mice, 6–8 weeks of age, were used for all experiments. The mice were housed in an air-conditioned animal house (26 ± 2 °C) and exposed to 10–12 h of light per day. Animals were maintained on a standard diet and had free access to water.

### Animal treatment

The animals were randomly divided into three groups for NaF treatment as follows:

Group I (NaF-male) was comprised of male mice given 100 mg/l NaF in water for 35 days. Group II (NaF-female) was comprised of female mice that were mated with the male mice that were not exposed to NaF. Then, only female mice with a vaginal plug were given 100 mg/l NaF in water for 48 h. Group III (Control) consisted of respective male and female animal groups, neither of which received NaF treatment.

All male mice were mated with females treated with PMSG (10 IU) and, 48 h later, with hCG (10 IU). Only female mice with vaginal plugs were determined to have successfully copulated, and this also was regarded as day 0.5 of pregnancy. Pregnant mice were bred continuously and eight-cell embryos and blastocysts were collected on days 2.5 and 3.5 of pregnancy, respectively. Liver samples and sperm were collected from five male mice from both groups (I, III).

### DNA isolation and global methylation determination

DNA from sperm and livers was isolated with the DNA easy kit (Qiagen) according to the manufacturer’s protocol and finally dissolved in 25–50 μl TE buffer. The concentration and quality of DNA were tested with a spectrophotometer (NanoDrop). Eight-cell embryos were treated in lysis solution (0.5 M EDTA, 2 mg/ml proteinase K, Ph 8) at 37 °C for 0.5 h and stored at −20 °C until use. Global methylation for sperm and liver was measured using a MethyFlash Methylated DNA Quantification Kit (Epigentek), an ELISA-based colorimetric assay. The assay was performed according to the manufacturer’s instructions. Relative quantification was assessed by normalizing samples against the positive control that was provided with the kit.

### Bisulfite treatment

Bisulfite treatment of genomic DNA was carried out as previously described (Zhu et al. 2008). Briefly, samples were denatured in 0.3 M NaOH at 37 °C for 15 min, mixed with 2 volumes of 2 % LMP (low melting point) agarose and pipetted into chilled mineral oil to form agarose beads. This was then treated with freshly made bisulfite solution (2.5 M sodium metabisulfite, Merck; 125 mM hydroquinone, Sigma; pH 5.0) at 50 °C for 16 h in the dark. The reaction was stopped by equilibrating the beads three times with 1 ml of TE buffer. Following desulfonation in 0.5 ml 0.3 M NaOH for 0.5 h, the beads were washed with TE buffer and H_2_O, respectively, and stored at −20 °C.

### PCR amplification

To avoid the preferential amplification of methylated or unmethylated sequences from a mixed population of starting molecules, nested PCR was performed using bisulfite-treated DNA in the first round (12.5 μl) and 2 μl of this PCR product as a template in the second round (25 μl). All reactions contained 0.4 mM primers, 0.2 mM dNTPs, 50 mM KCl, 10 mM Tris–HCl, 1.5 mM MgCl_2_, and 1.25 units of ExTaq hotstart polymerase (TaKaRa). PCR was performed on an ABI-9700 using the following program. The first round: 1 time at 94 °C for 6 min, 35 times at 94 °C for 1 min, annealing temperature (AT) for 2 min, 72 °C for 3 min and 1 time at 72 °C for 5 min; the second round: 1 time at 94 °C for 5 min, 30 times at 94 °C for 40 s, AT for 45 s, 72 °C for 50 s, 1 time at 72 °C for 5 min. The primers used for bisulfite sequencing were synthesized as previously described (Li et al. 2004; Lucifero et al. 2002; Okada et al. 2010) and these are listed in Table 1.

**Table 1:** Primers used for bisulfite sequencing

Gene	Primer sequence (5′–3′)	Size (bp)	Annealing temperature
H19	F1:GAGTATTTAGGAGGTATAAGAATT	423	45 °C
	R1:ATCAAAAACTAACATAAACCCCT		
	F2:GTAAGGAGATTATGTTTATTTTTGG		
	R2:CCTCATTAATCCCATAACTAT		
Peg3	F1:TGATAATAGTAGTTTGATTGGTAGGG	444	45 °C
	R1:TAATTCACACCTAAAACCCTAAAACC		
	F2:TTTTGTAGAGGATTTTGATAAGGAGG		
	R2:AAATACCACTTTAAATCCCTATCACC		
Rasgrf1	F1:AAGATAGTTTAGATATGGAATTTTGG	221	60 °C
	R:ATAATACAACAACAACAATAACAATC		
	F2:GATTTTTTAGAGAGTTTATAAAGTTAG		
Line1	F1:GTTAGAGAATTTGATAGTTTTTGGAATAGG	250	56 °C
	R1:CCAAAACAAAACCTTTCTCAAACACTATAT		
	F2:TAGGAAATTAGTTTGAATAGGTGAGAGGT		
	R2:TCAAACACTATATTACTTTAACAATTCCCA		

### DNA sequencing and restriction analysis of PCR products

The PCR products from three independent amplifications were gel-purified using the Wizard SV Gel and PCR Clean-Up System (Promega). Then, purified fragments were cloned into a T vector (TaKaRa). The positive clones confirmed by PCR were sequenced using an automatic sequencer (ABI PRISM-77). A portion of the purified fragments was digested with appropriate TaqI restriction enzymes (New England Biolabs). The digested fragments were electrophoresed on 3.0 % agarose gels.

### Semi-quantitative RT-PCR

Total RNA were isolated from 15 to 30 grouped embryos using the RNeasy Micro Kit (Qiagen). First-strand cDNA synthesis was carried out using PrimeScript reverse transcriptase (TaKaRa). PCR was performed on an ABI-9700 using the following program: 1 time at 94 °C for 6 min, 40 times at 94 °C for 30 s, 57 °C for 30 s, 72 °C for 40 s and 1 time at 72 °C for 5 min. The primers used for RT-PCR are given in Table 2. After PCR amplifications, each reaction mixture was separated by 2 % agarose gel electrophoresis. The intensity of each band was quantified and the relative intensity against *Gapdh* was calculated by using Gel Doc System (Bio-Rad).

**Table 2:** Primers used for RT-PCR

Gene	Primer sequence (5′–3′)	Size (bp)	Annealing temperature
H19	F: GTATGCCCTAACCGCTCAGTC	136	57 °C
	R: CCAACCTCCCTCCCTAGAAAC		
Peg3	F: TCAATGACCTCACAAGCCACCAG	261	57 °C
	R: CGGGCAACAGAGCGATGAAAGC		
Gapdh	F: TCTTGGGCTACACTGAGGAC	126	57 °C
	R: CATACCAGGAAATGAGCTTGA		

### Statistical analysis

The methylation rates in each group were expressed as mean ± standard error from bisulfite sequencing. Relative transcripts of each gene were expressed as mean ± standard error. All data were analyzed by one-way ANOVA, and the difference between groups was determined using the Student’s *T* test (*p* < 0.05 was considered statistically significant).

## Results

### NaF exposure to female mice during pregnancy disrupts *H19* DNA methylation in the embryo

**Fig. 1:**
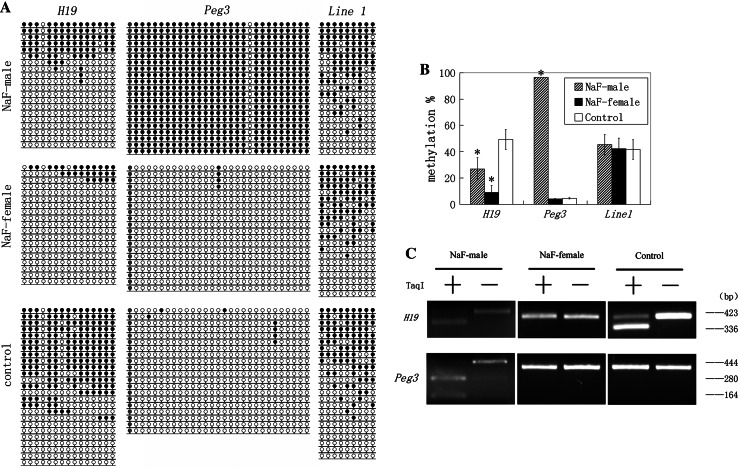
Methylation status of *H19*, *Peg3,* and *Line1* in embryos in NaF-male, NaF-female, and control groups. NaF-male mice were given 100 mg/l NaF in water for 35 days. NaF-female mice with a vaginal plug were given 100 mg/l NaF in water for 48 h, after mating with male mice not exposed to NaF. Control animals did not receive NaF treatment. **a** Methylation profiles assayed by a bisulfite sequencing assay. *Each line* represents an individual clone allele. *Each circle* within the *row* represents a single CpG site (*open* and *closed circles* represent unmethylated and methylated CpGs, respectively). **b** Statistical analysis of methylation in three groups. Bisulfite sequencing (**a**) was used to plot the percent of methylated CpGs of the total number of CpG sites. Data are presented as mean ± standard error. (*p < 0.05, compared with controls). The same illustration was applied to the following figures. **c** Overall methylation profiles of *H19* and *Peg3* as revealed by bisulfite restriction analysis. The same bisulfite-treated DNA sample used for sequencing was digested with restriction enzymes. Restriction enzymes cleaved only if the recognized TaqI site is methylated. −, undigested PCR products; +, digested PCR products. Sizes of digested fragments are indicated on the right. Digestion of PCR products with TaqI enzyme produced cleaved and uncleaved products, suggesting that PCR products include methylated and unmethylated templates

DNA methylation of *H19*, *Peg3,* and *Line1* was examined in NaF-treated male, NaF-treated female, and respective control groups. The results of bisulfite sequencing and bisulfite restriction analysis are summarized in Fig. 1. The data show that DNA methylation of *H19* was almost entirely lost in the NaF-treated female group compared with the control group (Fig. 1a). Methylation of *H19* in NaF-treated female and control groups were 9.12 ± 5.28 and 49.33 ± 7.60 %, respectively, and the differences between them were significant (*p* < 0.001) (Fig. 1b). PCR products of *H19* in NaF-treated females were undigested, unlike those observed in the female untreated control group (Fig. 1c). The cleaved and uncleaved products refer to methylated and unmethylated templates, respectively.

The pattern and degree of methylation in *Peg3* and *Line1* were comparable between NaF-treated females and control females (Fig. 1a). *Peg3* methylation in NaF-treated females and control females was 4.14 ± 0.32 and 4.66 ± 0.45 %, respectively. *Line1* methylation in NaF-treated females and control females groups was 42.33 ± 7.99 and 41.67 ± 7.42 %, respectively. These differences were not significant (Fig. 1b). Furthermore, bisulfite restriction analysis did not clearly reveal the significant difference of *Peg3* between NaF-female and control groups (Fig. 1c).

### NaF treatment of male mice before copulation disrupts DNA methylation of *H19* and *Peg3* in the developing embryo

When female mice were mated with male mice treated with NaF in water for 35 days, DNA methylation was significantly decreased in *H19* and increased in *Peg3* compared to *H19* and *Peg3* in male mice which did not receive NaF treatment. NaF treatment of male mice did not affect methylation of *Line1* in the developing embryo. The restriction pattern of *H19* was similar between NaF-treated male mice and the control male group (Fig. 1c), and bisulfite sequencing revealed that the *H19* methylation in the NaF-treated male group was 27.00 ± 8.54 %, which was significantly lower than that observed in the control male group (*p* < 0.05) (Fig. 1a, b). *Peg3* methylation in NaF-treated male mice was 96.55 ± 0 %, significantly higher than that observed in the control male group (*p* < 0.001) (Fig. 1a, b). The majority of *Peg3* PCR products in NaF-treated mice were digested, unlike those observed in the control male group (Fig. 1c).

### NaF treatment of male mice before copulation increase the expression of *H19* in blastocysts

**Fig. 2:**
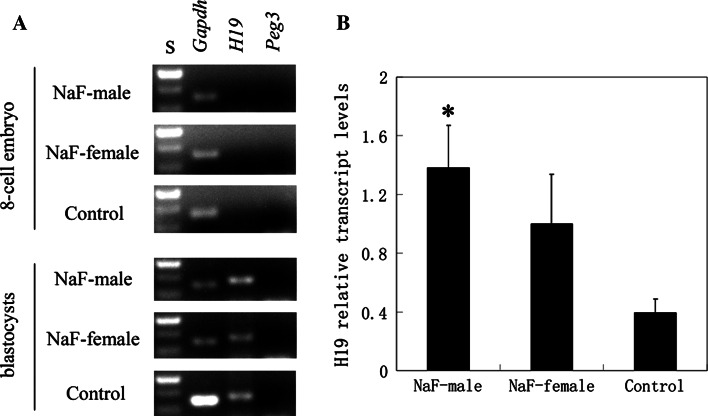
H19 and Peg3 expression in 8-cell embryo and blastocysts from NaF-treated and control mice. **a** Agarose gel electrophoresis. S, 100 bp ladders. **b** *H19* relative transcript levels. Transcripts were normalized against the *Gapdh* control. *Each column* indicates the mean ± standard error (*p < 0.05, compared with controls)

The expression of *H19* and *Peg3* was examined in 8-cell embryos and blastocysts from all mouse groups (See Fig. 2). *H19* was expressed at the blastocyst stage, whereas Peg3 was not. NaF treatment of male mice before copulation significantly increased *H19* expression in blastocysts. At the 8-cell embryo stage, *H19* and *Peg3* expression could not be detected by RT-PCR.

### NaF may not significantly affect DNA methylation of sperm and liver

**Fig. 3:**
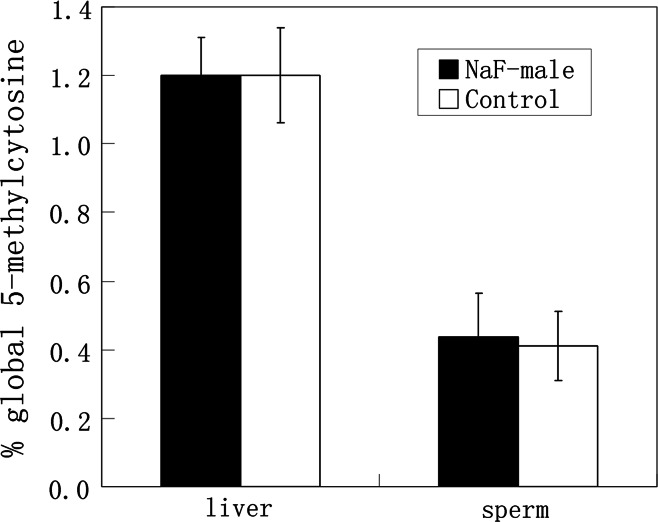
5-methylcytosine in genomic DNA of sperm and liver from NaF-treated male and control groups were measured using a MethylFlash Methylated Quantification kit

**Fig. 4:**
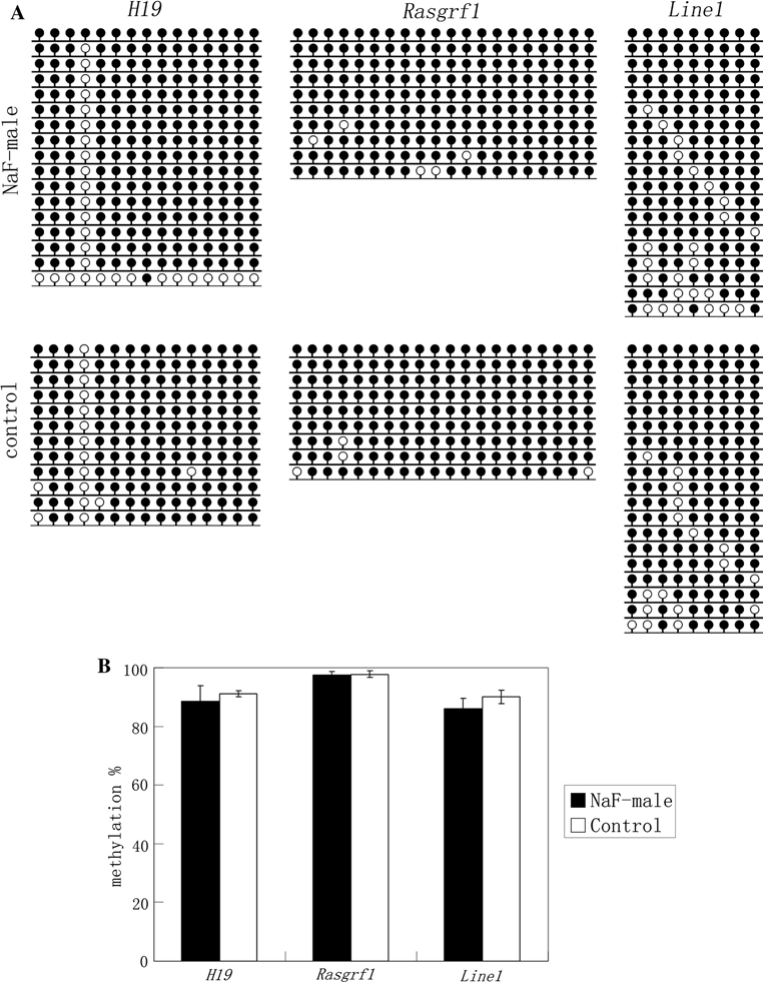
Methylation status of *H19*, *Rasgrf1,* and *Line1* on sperm in NaF-male and control groups. **a** Methylation profiles assayed by the bisulfite sequencing assay. **b** Statistical analysis of methylation in two groups. Bisulfite sequencing (**a**) was used to plot the percent of methylated CpGs of the total number of CpG sites

**Fig. 5:**
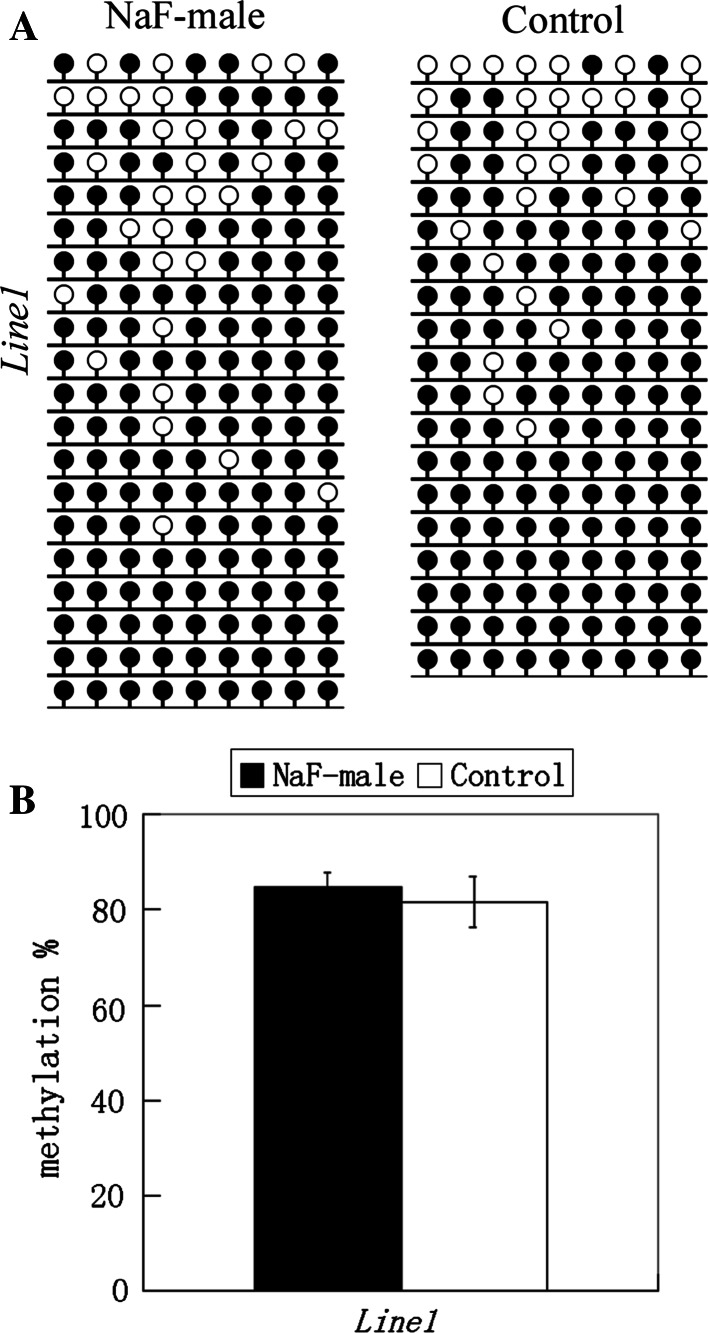
Methylation status of *Line1* on liver in NaF-male and control groups. **a** Methylation profiles assayed by the bisulfite sequencing assay. **b** Statistical analysis of methylation in two groups. Bisulfite sequencing (**a**) was used to plot the percent of methylated CpGs of the total number of CpG sites

Global DNA methylation in sperm and liver were examined in NaF-treated male mice and their corresponding control group. Using an ELISA-based colorimetric assay, 5-methylcytosine in genomic DNA was measured (Fig. 3). In sperm from NaF-treated male mice and the control male group, 5-methylcytosine was 0.44 ± 0.13 and 0.41 ± 0.10 %, respectively, and 5-methylcytosine in the livers of those treatment and control groups was 1.20 ± 0.11 and 1.20 ± 0.14 %, respectively. Furthermore, DNA methylation of *H19*, *Rasgrf1,* and *Line1* in sperm and *Line1* methylation in liver were examined using bisulfite sequencing (Figs. 4, 5), and no significant differences were observed in all examinations. Data indicate that NaF may not significantly affect DNA methylation in the sperm and liver of male mice.

## Discussion

NaF not only cause pathological changes in a variety of tissues and organs, but also causes cancer and malformation of the reproductive system. The mechanism of NaF reproductive toxicity is not well understood, although DNA methylation is known to be a particularly important epigenetic modification mechanism. Abnormal DNA methylation is also thought to be involved in the etiology of complex diseases (Fraga et al. 2005). In the present study, the effects of NaF on DNA methylation in the embryo and in sperm and the liver were investigated. *H19*, *Peg3,* and *Rasgrf1,* which are imprinted genes, were selected for study because of their important biological functions and their characteristic phenotypes. The methylation of the *Line1* repeated sequence was also investigated as an indicator of genome methylation. Data indicated that NaF treatment disrupted DNA methylation of *H19* and *Peg3* in early embryonic stages of the mouse. However, there were no significant changes in DNA methylation in sperm and liver of male mice treated with NaF. Because gene expression is affected by DNA methylation, we then investigated the effect of NaF on the expression of *H19* and *Peg3* in 8-cell embryos and in the blastocyst. NaF treatment of male mice before copulation significantly increased the expression of *H19* in blastocysts, whereas *H19* expression was not detected in 8-cell embryos. These findings indicate that NaF can disrupt DNA methylation of *H19* and further affect *H19* expression.

Oral ingestion of NaF in water is usually the major source of fluoride toxicosis. The mice treated with 100 mg/l NaF in water were used to model this toxicity. Pregnant female mice and male mice were treated for 48 h and 35 days, respectively. Many studies of laboratory animals indicate that adverse reproductive and developmental effects occur at high fluoride concentrations and that various injurious effects are associated with an exposure of 100 mg/l NaF in drinking water (Chlubek et al. 2003; Shanthakumari et al. 2004). The experimental period was aligned with the cycle of mouse spermatogenesis. For gonad stem cells to mature into sperm, 35 days are required. Within these 35 days, the spermiogonium stage lasts 8 days; meiosis lasts 13 days; and sperm formation needs 14 days. Finally, 5 days are needed for sperm to reach the tail of epididymis for ejaculation (Zhu et al. 2006).

In the processes of imprint erasure, establishment and maintenance are vulnerable to errors. Methylation imprints could not be re-established in the postzygotic embryos, once they were lost (Chang et al. 2006; Sato et al. 2007). Our results suggest that *H19* is demethylated in embryos from NaF-treated pregnant mice, resulting in an undermethylation pattern in the embryos. This is consistent with early reports demonstrating that *H19* is a target for environmental insults (Doherty et al. 2000; Joss-Moore and Lane 2012). In contrast, *Peg3* imprints are not affected by NaF exposure during pregnancy. This discrepancy may be due to the fact that *Peg3* methylation can be erased after fertilization (Liu et al. 2008). However, in this present study, we did not investigate whether NaF ingestion during pregnancy affected re-establishment of the *Peg3* imprint in the morula or blastocyst stage. *Line1* methylation, as an indicator of genome methylation, is not affected by NaF ingestion during pregnancy. Our data support the idea that NaF can cross the placental barrier and interact directly with pre-implantation embryos. NaF can disrupt maintenance of the *H19* imprint during pre-implantation development, while genomic DNA methylation can be normally reprogrammed. Thus, *H19* methylation is a target for NaF reproductive toxicity.

We next investigated the effects of NaF on DNA methylation of sperm and whether this effect can be transferred to early embryos. Although fluoride can cross the blood-testis barrier to inhibit testicular spermatogenesis and decrease sperm capacitation (Dvorakova-Hortova et al. 2008; Sarkar et al. 2006), NaF did not significantly affect DNA methylation in *H19*, *Rasgrf1*, *Line1* and genome methylation during spermatogenesis. Of note, *H19* and *Peg3* were abnormally methylated in the embryo when NaF-treated male mice sperm were the source of oocyte fertilization. Data show that NaF does not directly affect DNA methylation in the sperm, but NaF can potentially affect regulation mechanisms of methylation imprints (this was beyond the scope of this investigation). Also, sperm that were not directly damaged may “carry” or transmit this insult to early embryos, giving rise to abnormal methylation imprints. In this case, *Peg3* hypermethylation caused by NaF may result from early events of remethylation, because *Peg3* is completely unmethylated in sperm and 2-cell embryos. In addition, we investigated the effect of NaF on DNA methylation in the liver. NaF did not appear to effect genome methylation in the liver, where DNA methylation reprogramming is not found.

In summary, we propose that NaF can harm DNA methylation of imprinted genes in embryos, but clearly, genome methylation can withstand some adverse effects of NaF and maintain balance. Our data have provided essential preliminary data for further study of potential NaF-induced reproductive toxicity. At a minimum, our data suggest that the use of fluoride in dental care products may require re-evaluation for pregnant women and men who plan to father children.
